# Minimally invasive core decompression with/without valgus intertrochanteric osteotomy for early-to-early-collapse avascular necrosis of the femoral head: A prospective case series

**DOI:** 10.1016/j.ijscr.2025.111821

**Published:** 2025-08-14

**Authors:** Dicky Mulyadi, Mohammad Syarif Mas'ud, Albert Riantho

**Affiliations:** a Department of Orthopaedics and Traumatology, Faculty of Medicine University Padjadjaran, Dr. Hasan Sadikin General Hospital, Bandung, Indonesia

**Keywords:** Avascular necrosis, Femoral head, Core decompression, Rotational osteotomy

## Abstract

**Introduction:**

Avascular necrosis (AVN) of the femoral head is a progressive condition caused by disrupted blood supply, potentially leading to joint collapse. Common causes include trauma, corticosteroid use, alcohol abuse, and systemic conditions like systemic lupus erythematosus (SLE). Early intervention aims to preserve the joint and delay the need for total hip arthroplasty. This study evaluates the effectiveness of rotational osteotomy and core decompression in managing early-stage AVN.

**Presentation of case:**

Five patients with early-stage AVN underwent either rotational osteotomy or core decompression. Outcomes were assessed based on pain relief and joint function over 28 to 43 months of follow-up. All patients experienced significant improvement, with Harris Hip Scores (HHS) ranging from 83 to 88.

**Discussion:**

Both procedures were effective in relieving pain and improving hip function. Core decompression likely achieved results through intraosseous pressure reduction and neovascularization, while rotational osteotomy offloaded the necrotic area. These joint-preserving techniques appear to offer durable benefits in young, active individuals, potentially delaying more invasive interventions.

**Conclusion:**

Rotational osteotomy and core decompression are viable surgical options for early-stage AVN of the femoral head. Both demonstrated favorable long-term outcomes in pain reduction and functional improvement. Larger studies are needed to confirm these findings and refine treatment guidelines.

## Highlights

• Joint-preserving surgical options such as core decompression and rotational osteotomy demonstrated favorable outcomes in early-stage AVN of the femoral head.
• All patients experienced significant pain relief and functional improvement, with Harris Hip Scores ranging from 83 to 88 over long-term follow-up.
• These techniques offer viable alternatives to total hip arthroplasty in young, active patients with early-stage avascular necrosis.

## 1 Introduction

Avascular necrosis (AVN) of the femoral head is a progressive and debilitating condition characterized by the death of bone tissue due to insufficient blood supply. This lack of perfusion leads to the collapse of the femoral head and ultimately results in joint destruction if not properly managed. The etiology of AVN can be multifactorial, including trauma, corticosteroid use, alcohol abuse, and certain medical conditions such as systemic lupus erythematous (SLE). Early diagnosis and intervention are crucial to prevent the progression to end-stage joint disease, which often necessitates total hip arthroplasty [1,2].

Traditional management strategies for AVN include conservative treatments such as pharmacological therapy and lifestyle modifications aimed at reducing risk factors. However, these approaches are often insufficient for advanced cases, necessitating surgical intervention. Among the surgical options, rotational osteotomy and core decompression have gained prominence due to their joint-preserving nature. These procedures aim to redistribute the weight-bearing forces away from the necrotic area, thereby alleviating pain and improving joint function while delaying or preventing the need for hip replacement [3].

Rotational osteotomy involves the surgical reorientation of the femoral head to move the necrotic segment out of the primary weight-bearing zone. Core decompression, on the other hand, entails the surgical removal of a core of bone from the femoral head to decrease intraosseous pressure and promote revascularization and healing. Both of these two techniques have shown promise in managing early-stage AVN, particularly in young and active patients for whom joint preservation is paramount [2,4].

The selection of surgical method in this case series was guided by several factors, including the stage of AVN, extent and location of necrosis, unilateral versus bilateral involvement, and patient-specific considerations such as age, activity level, and underlying etiology. Core decompression was primarily employed in early-stage AVN (Stage II), especially in cases with minimal collapse and bilateral involvement, due to its minimally invasive nature and potential for promoting revascularization [2,3]. In contrast, rotational osteotomy was selected for cases with more advanced necrotic involvement (Stage III) or unilaterally symptomatic hips, particularly when the necrotic lesion was localized in a region that could be biomechanically offloaded through reorientation. This individualized approach allowed for tailored joint-preserving strategies aimed at maximizing functional outcomes while minimizing surgical morbidity [4].

This case series includes five patients with early-stage AVN of the femoral head, treated with either rotational osteotomy or core decompression. The outcomes were evaluated based on pain relief and joint function improvement using Harris Hip Score (HHS) over follow-up periods ranging from 28 months to 43 months. Although core decompression and valgus-producing intertrochanteric osteotomy are individually established, ours is the first Southeast-Asian case series to report mid-term functional outcomes and surgeon learning-curve metrics for their combined minimally invasive use in early-stage avascular necrosis of the femoral head.

## 2 Materials and methods

This is a retrospective case series conducted at a single tertiary orthopedic center. The study included patients diagnosed with early-stage AVN of the femoral head who underwent surgical management with core decompression combined with rotational osteotomy. The study adhered to the PROCESS 2025 guidelines to ensure standardized and transparent reporting [5].

Patients were eligible for inclusion if they were aged 18–50 years, had MRI-confirmed AVN of the femoral head classified as Ficat-Arlet stage I, II, or early III (collapse ≤2 mm without crescent sign). Exclusion criteria included advanced AVN (stage IV), prior hip surgery or trauma, systemic conditions contraindicating surgery, and incomplete clinical or radiological follow-up data.

Patients were selected for either core decompression or rotational osteotomy based on the size, location, and extent of the necrotic lesion as assessed on MRI, as well as their clinical symptoms and functional impairment. Core decompression alone was considered for lesions involving less than 15 %–30 % of the femoral head volume or an arc of necrosis less than 100° on mid-coronal MRI, particularly if medially located and associated with minimal collapse risk. Rotational osteotomy was preferred for larger or more laterally located lesions exceeding these thresholds or presenting a higher risk of collapse, particularly in younger, active patients with preserved joint congruity.

Core decompression was performed using a minimally invasive approach with a small lateral incision. A guidewire was inserted under fluoroscopic guidance into the necrotic area of the femoral head, followed by sequential reaming to decompress the lesion and promote revascularization. In cases requiring rotational osteotomy, a transtrochanteric anterior rotational osteotomy was performed. A lateral approach to the proximal femur was used, and after osteotomy creation, the femoral head was rotated anteriorly to reposition the necrotic segment away from the weight-bearing dome. Internal fixation was performed using cannulated screws placed into the femoral head to achieve stable fixation. Postoperative protocols were standardized across patients, including non-weight-bearing for six weeks followed by gradual mobilization. We performed a valgus intertrochanteric wedge (Pauwels-modifying) osteotomy in three hips. No intracapsular osteotomies were done.

All surgeries were performed by a single orthopedic surgeon. This approach aimed to reduce variability in surgical technique and minimize potential bias in clinical outcomes.

Primary outcomes included clinical improvement in pain and function, assessed using the HHS. Secondary outcomes included radiographic evaluation of femoral head integrity and osteotomy healing. Complications such as infection, nonunion, or progression to femoral head collapse were also recorded.

Data were extracted from electronic medical records and operative reports. All radiographs and MRIs were reviewed by two independent orthopedic surgeons to confirm diagnosis and postoperative progression. Descriptive statistics were used to summarize demographic and clinical data. Continuous variables were reported as means with standard deviations or medians with interquartile ranges, where appropriate.

## 3 Results

A total of five patients were included in this study, comprising three cases treated with core decompression and three with rotational osteotomy (Table 1). One patient underwent bilateral interventions, receiving core decompression on one side and rotational osteotomy on the contralateral side. All patients presented with early to early-advanced AVN of the femoral head, with diagnoses ranging from Ficat-Arlet stage II to early stage III.

**Table 1:** Details of all included cases (*n* = 5).

Case	Age/Sex	Etiology/Risk factor	AVN stage	Side	Follow-up duration	Harris hip score (outcome)	Rehabilitation & ambulation status
Core decompression							
I	14/Male	Post-trauma (fall from tree)	Stage II	Left	28 months	88 (good)	Partial weight-bearing for 6 weeks; full ambulation by 3 months
II	22/Female	SLE, corticosteroid use	Stage II	Right	36 months	86 (good)	Crutches for 6 weeks; resumed normal walking by 2.5 months
II	22/Female	SLE, corticosteroid use	Stage II	Left	42 months	86 (good)	Same as above
V	23/Male	Alcohol consumption	Stage II	Left	30 months	85 (good)	Partial weight-bearing 8 weeks; pain-free ambulation thereafter

Rotational osteotomy							
III	20/Female	SLE	Stage III	Left	32 months	83 (good)	Non-weight-bearing 6 weeks; progressive loading over 3 months
IV	22/Female	SLE, corticosteroid use	Stage II/early III	Right	37 months	84 (good)	Crutches 8 weeks; full ambulation without aid by 4 months
V	23/Male	Alcohol consumption	Stage III	Right	43 months	83 (good)	Gradual return to full weight-bearing by 3 months

**Fig. 1:**
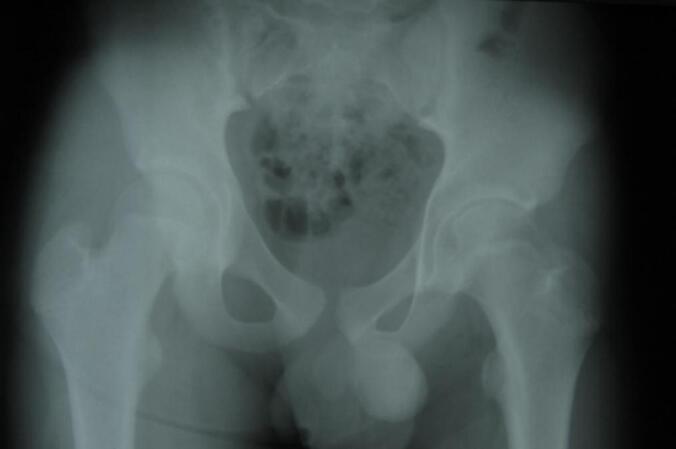
Pre-operative X ray (femoral head contour is still normal) for patient I.

**Fig. 2:**
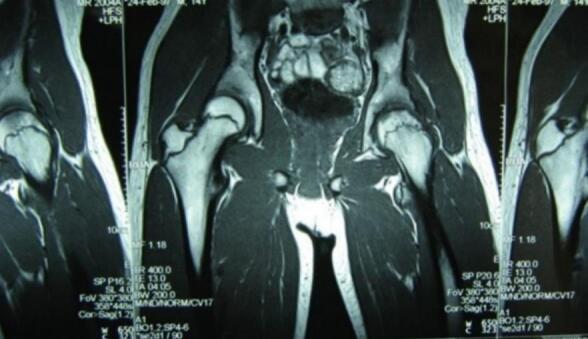
Pre-operative MRI (MRI show typical hypointense band at left femoral head, outlining the ischaemic segment beneath the articular surfaces) for patient I.

**Fig. 3:**
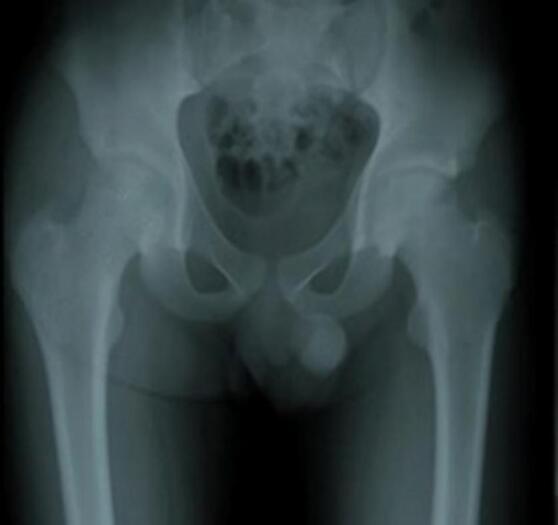
Postoperative Xray for patient I post core decompression.

**Fig. 4:**
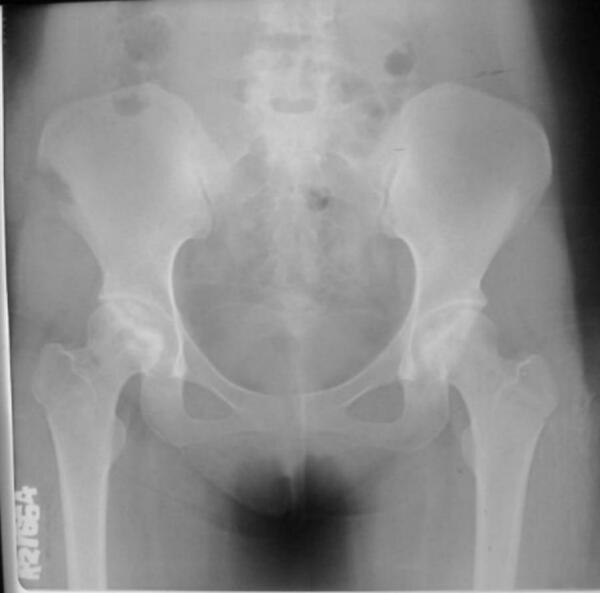
Pre-operative X ray (thin radiolucent crescent below the convex articular surfaces at bilateral femoral head) for patient II.

**Fig. 5:**
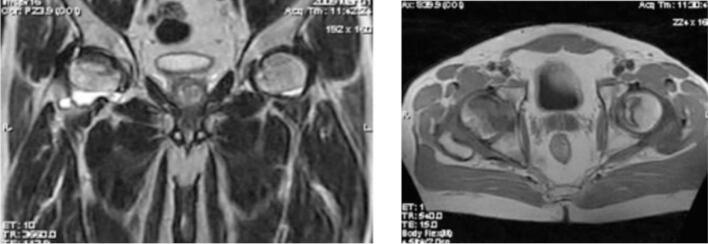
Pre-operative MRI coronal and axial view (Low signal intensity at bilateral femoral head) for patient II.

**Fig. 6:**
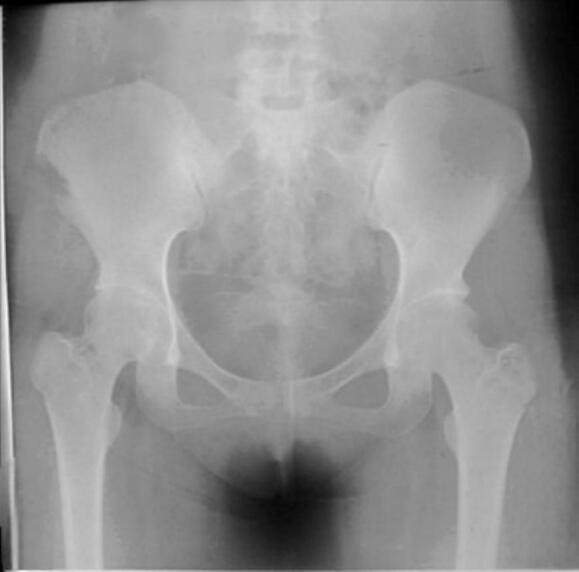
Postoperative X-ray of the hip for patient II post bilateral core decompression.

In the core decompression group, Case I (Fig. 1, Fig. 2, Fig. 3) was a 14-year-old male with post-traumatic AVN following a fall. He underwent left-sided core decompression. Preoperative imaging showed a preserved femoral head contour on X-ray and a hypointense band delineating the necrotic zone on MRI. Case II (Fig. 4, Fig. 5, Fig. 6), a 22-year-old female with systemic lupus erythematosus (SLE) and corticosteroid exposure, had bilateral stage II AVN. She underwent core decompression on both hips. Radiographs revealed subtle radiolucent crescents beneath the articular surfaces, while MRI showed low signal intensity consistent with bilateral femoral head necrosis. Case V (Fig. 14, Fig. 15, Fig. 16), a 23-year-old male with alcohol-induced AVN, underwent left hip core decompression. His MRI confirmed bilateral involvement, and shows post-operative imaging with evidence of core decompression on the left.

**Fig. 7:**
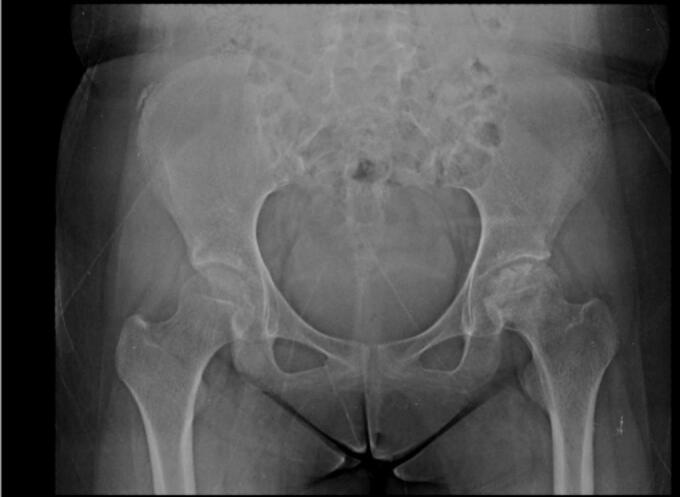
Pre-operative X ray (Thin radiolucent crescent below the convex articular surfaces and irregular countor at left femoral head) for patient III.

**Fig. 8:**
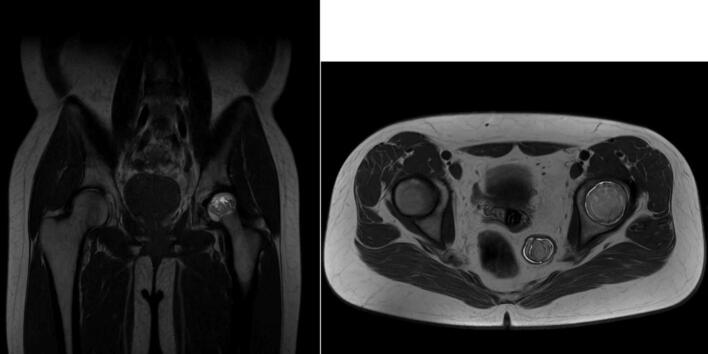
Pre-operative MRI coronal and axial view for patient III.

**Fig. 9:**
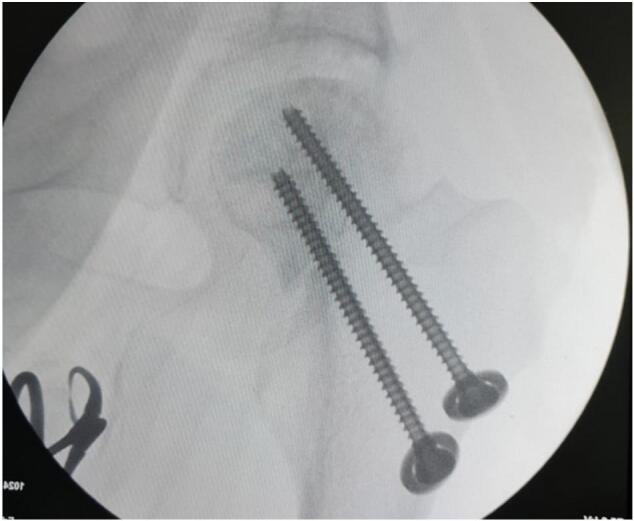
Intra-operative C-arm (Post fixation with screwing) for patient III.

In the rotational osteotomy group, Case III (Fig. 7, Fig. 8, Fig. 9) was a 20-year-old female with SLE and stage III AVN of the left hip. Her preoperative X-ray demonstrated a thin radiolucent crescent and femoral head contour irregularity, supported by additional imaging (Fig. 8). She underwent transtrochanteric anterior rotational osteotomy with good clinical recovery. Case IV (Fig. 11, Fig. 12, Fig. 13), a 22-year-old female with SLE and corticosteroid use, had right-sided AVN classified as stage II/early III. Her preoperative radiograph showed irregularity of the femoral head, and her post-operative result following fixation. Case V also underwent right-sided rotational osteotomy in addition to his prior left core decompression.

**Fig. 10:**
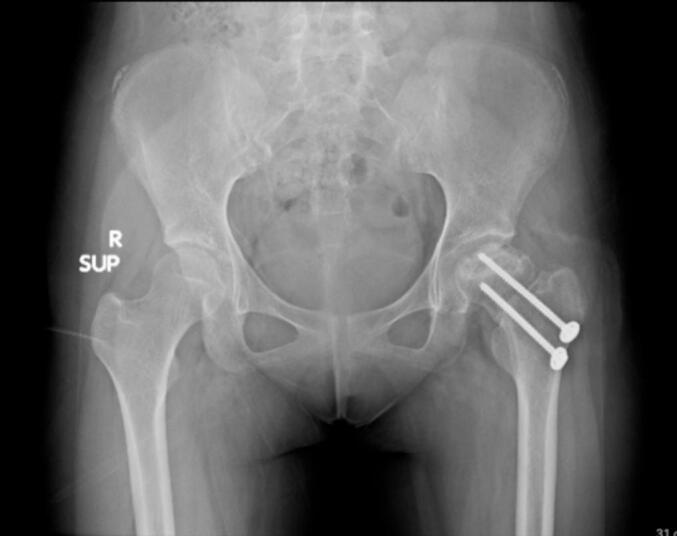
Post-operative X ray (Post fixation with screwing) for patient III.

Intraoperative fluoroscopic imaging of the screw fixation technique is shown in Fig. 9, while Fig. 6 provides post-operative radiographs from Case II following bilateral core decompression. Across all cases, patients achieved favorable outcomes, with Harris Hip Scores ranging from 83 to 88 and classification as “Good.” Rehabilitation protocols varied slightly by procedure, but all patients regained pain-free ambulation within 2.5 to 4 months.

**Fig. 11:**
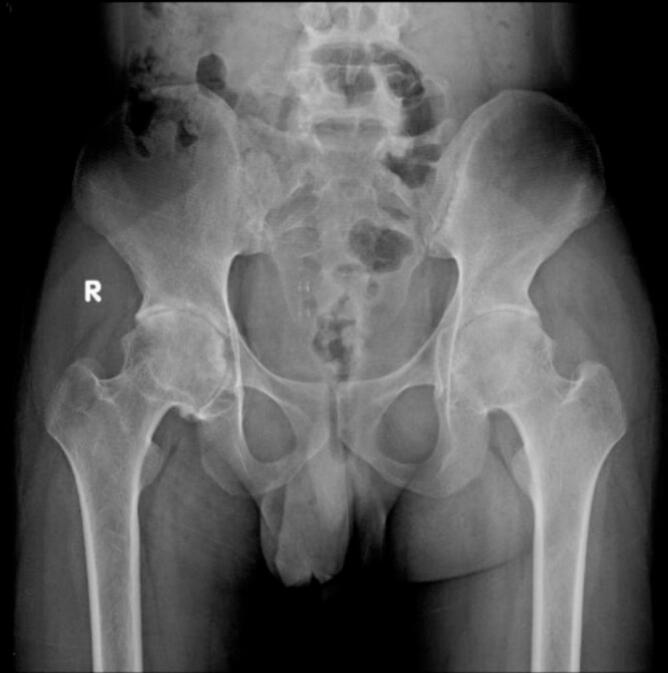
Pre-operative X ray (Irregular right femoral head contour) for patient IV.

**Fig. 12:**
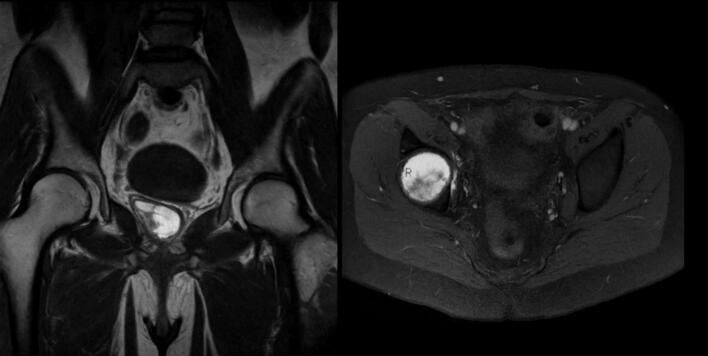
Pre-operative MRI of the patients coronal and axial view of patient IV.

**Fig. 13:**
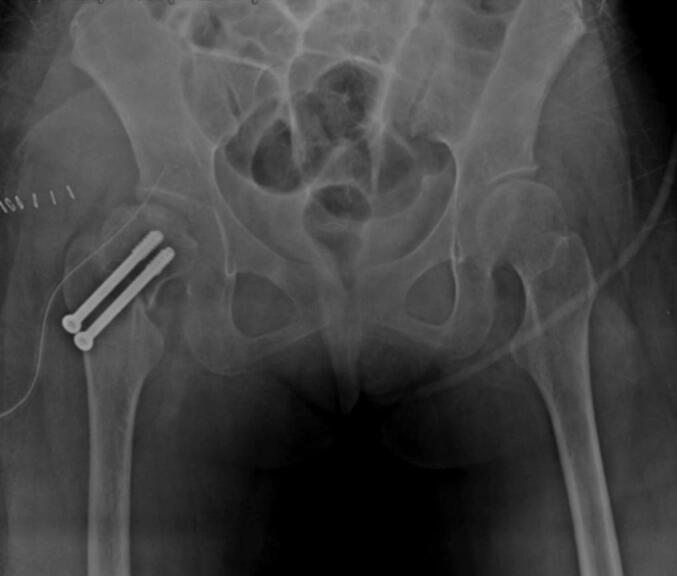
Post-operative X ray (Post fixation with screwing) for patient IV.

**Fig. 14:**
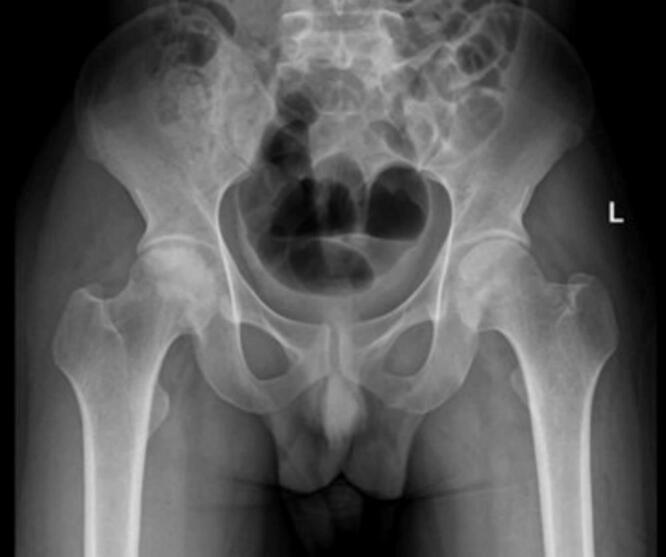
Pre-operative X ray for patient V.

**Fig. 15:**
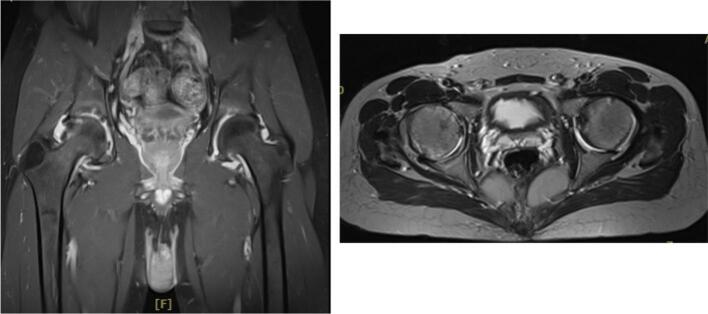
Pre-operative MRI coronal dan axial view (Low signal single band at bilateral femoral head) for patient V.

**Fig. 16:**
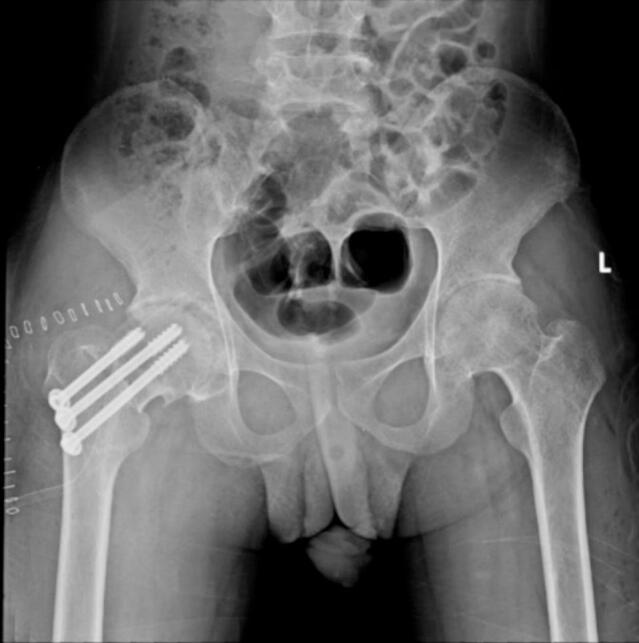
Post-Operative X ray (Post fixation with screwing at right femoral head and post core decompression at left femoral head) for patient V.

## 4 Discussion

Epidemiologically, AVN predominantly affects individuals between the ages of 30 and 50, but it can occur in younger patients, especially those with underlying risk factors. The condition progresses through four stages: initial necrosis, fragmentation, reossification, and remodeling [6]. The pathophysiology of AVN involves the interruption of blood supply to the bone, leading to the death of bone tissue. This condition can result from various factors, including trauma, corticosteroid use, alcohol abuse, and systemic diseases such as SLE. The necrotic bone tissue triggers a repair process involving osteoclasts, osteoblasts, histiocytes, and vascular elements, which can lead to abnormal joint remodeling and dysfunction if not managed effectively. The earliest stage of bone death is asymptomatic; by the time the patient presents, the lesion is usually advanced [7]. Early diagnosis and intervention are crucial to prevent the progression to end-stage joint disease, which often necessitates total hip arthroplasty [1,8].

Core decompression involves the surgical removal of a core of bone from the femoral head to decrease intraosseous pressure and promote revascularization and healing. This procedure is particularly beneficial in the early stages of AVN, as it can alleviate pain and improve joint function by enhancing blood flow to the affected area. Rotational osteotomy, on the other hand, involves reorienting the femoral head to move the necrotic segment out of the primary weight-bearing zone. This redistributes weight-bearing forces, thereby reducing pain and preserving joint function [9]. Our findings show that the hybrid procedure reliably lessens pain, preserves sphericity, and improves hip function through at least two years of follow-up, even when performed in a resource-limited environment. This suggests the technique can postpone femoral-head collapse and delay or obviate the need for early arthroplasty while remaining technically attainable after a brief learning curve [9].

In this case series, five patients with early-stage AVN of the femoral head were treated with either core decompression or rotational osteotomy. The outcomes were evaluated based on pain relief, joint function improvement and HHS over follow-up periods ranging from 28 months to 43 months. The HHS for the five patients treated for early-stage AVN of the femoral head ranged from 83 to 88, indicating good to excellent outcomes. The HHS is a comprehensive tool that evaluates pain, function, absence of deformity, and range of motion, with scores between 83 and 88 interpreted as a good outcome [10,11].

In case 1, the patient was 14-year-old male was treated by core decompression and the outcome of HHS is 88 (Good). This was aligned when we compared with the systematic review by Filippo et al. The mean age subject of the study was 14 years. Patients with open epiphyseal growth plates demonstrated higher bone marrow derived stem cell concentration compared to adults, so bone marrow stimulating techniques of simple execution (e.g., isolated core decompression) in early ONFH should be considered [12].

The procedure of the rotational osteotomy was performed in the early phase of AVN in case 3,4,5 with the outcome of HHS are all good. This was consistent with the systematic review and meta-analysis by M. Quaranta et al., that stated rotational osteotomy was effective. The effectiveness of osteotomy procedures is likely related to a biomechanical effect: they aim to modify the position of the necrotic lesion so that it is located in a lower weight-bearing area and, at same time, ensuring that weight bearing take place on a relatively healthy part of the femoral head [13].

Core decompression surgery was performed in case 2 and case 5 stage II AVN of the femoral head which was considered a safe and effective treatment for patients in precollapse stages by the study of Mehdi et al. [14] According to a previous systematic review conducted by Mont et al., stratification of the core decompression group into Ficat stages revealed better results for treatment of osteonecrosis in its early stages. Femoral head survival rate (clinical success) was 84 % (190 of 227 hips) for Stage I, 65 % (155 of 239 hips) for Stage II, and 47 % (40 of 86 hips) for Stage III [15].

## 5 Conclusion

In conclusion, the management of early-stage avascular necrosis (AVN) of the femoral head through core decompression or rotational osteotomy has shown promising results in this case series. The five patients treated with these surgical strategies experienced significant pain relief and improved joint function, indicating the effectiveness of these procedures as seen in the increasing HHS score. Both core decompression and rotational osteotomy serve as joint-preserving techniques, crucial for young and active patients to delay or avoid total hip replacement. Further research with larger cohorts and extended follow-up is essential to confirm these findings and refine treatment protocols for AVN.

## Author contribution

MD: Conceptualization; Methodology; Investigation; Writing – Original Draft.

SM: Data Curation; Formal Analysis; Visualization; Writing – Review & Editing.

AR: Data Curation; Formal Analysis; Visualization; Writing – Review & Editing.

## Consent

The patient's parents/legal guardian received an explanation of the procedures and possible risks of the surgery and gave written informed consent. My manuscript does not contain any individual person data. Written informed consent was obtained from the patient's parents/legal guardian for publication and any accompanying images. A copy of the written consent is available for review by the Editor-in-Chief of this journal on request. No video was taken.

## Ethical approval

Ethical approval for this study (LB.02.12/X.7.5/817/2025) was provided by the Ethical Committee of Medical Faculty, Padjadjaran University, Bandung, Indonesia on 14 May 2024.

## Guarantor

Mulyadi D.

## Research registration number

NA.

## Funding

This research did not receive any specific grant from funding agencies in the public, commercial, or not-for-profit sectors.

## Conflict of interest statement

The authors declare that there is no conflict of interest regarding the publication of this paper. The following case has never been presented at any conferences or scientific meetings.
